# The role of discrete sample injection in trace mercury analysis by atomic fluorescence spectrometry

**DOI:** 10.1155/S1463924696000168

**Published:** 1996

**Authors:** P. B. Stockwell, W. T. Corns, N. Brahma

**Affiliations:** P S Analytical Ltd, Arthur House, Unit 3 Crayfields Industrial Estate Main Road Orpington, Kent BR5 3HP UK


Journal of Automatic Chemistry, Vol. 18, No. 4 (July–August 1996), pp. 153–162

### The role of discrete sample injection in trace mercury analysis by atomic fluorescence spectrometry

**P. B. Stockwell, W. T. Corns and N. Brahma**
*P S Analytical Ltd, Arthur House, Unit 3, Crayfields Industrial Estate, Main Road, Orpington, Kent BR5 3HP, UK*

*Coupling specific atomic fluorescence spectrometers to vapour generation techniques is a highly sensitive approach to the determination of trace levels of mercury. In many sample types the levels of the mercury content are extremely high and the matrix may have a deleterious effect on the measurement. This paper discusses the application of discrete sample injection techniques to broaden the range of analytes tested and the levels analysed. The limitation of linear dynamic range for fluorescence is the self-absorption effect. Reducing the effective sample size to below 100 µ litres allows a linear calibration up to 10 parts per million (ppm). This sample limitation, coupled to the software's ability to reset the sampling valve should the signal level exceed the maximum setting, ensures that levels of up to 100 ppm can be presented to the analyser. An additional advantage of the discrete sample injection applies to complex analytical samples, for example concentrated sulphuric acid. The effective dilution provided by this means overcomes any matrix interferences and quickly provides correct data. With proper care, the analytical range of the system described can extend over seven orders of magnitude from less than 1 part per trillion (ppt) through to 10 ppm.*

### Introduction

Over the past decade there has been considerable concern about the levels of heavy metals in the environment, especially mercury, arsenic, selenium and antimony. Since the authors became interested in this field, levels of mercury have received by far the most attention. Currently the legislation sets out the limits for mercury as the total content, whatever the form in which the mercury may be present. However, there is a pressing case for analysing the various species of mercury present; methylmercury, for example, is more than 1000 times more toxic than mercury in its inorganic forms. Recently Jones *et al.* [1] have suggested a simple gas chromatographical separation system, linked to a specific atomic fluorescence detector, to determine such species in a range of materials including soils, sludges and effluent.

In 1988, the reorganization of the UK water industry into privatized companies and a policing facility provided by the National Rivers Authority laboratories drew attention to the monitoring of mercury in drinking water. The levels required, and the sampling frequency which were dictated by the legislation, meant that a radically new direction for instrumentation was needed. The batch methods using atomic absorption detection available at this time provided neither the detection limits nor the throughput necessary.

Thompson and Godden [2] described an atomic fluorescence method for the measurement of mercury; Godden and Stockwell [3], using an available molecular fluorescence detector with subtle modifications, designed a simple but effective commercial variation of this with the additional potential for complete automation. In 1989, P S Analytical introduced the world's first fully automated mercury analyser based on these developments. Since then more than 20 commercial competitors have been introduced around the world. With each of these making various claims as to detection capabilities, it would seem to the analytical community that the determination of mercury at low levels is just a trivial matter. This is very far from the truth because at the levels required, often between $10^{-10}$ and $10^{-12}$ g litre, it is very difficult to get representative samples and reproducible results. With care to the sampling and methodology, levels below 1 ppt can be measured.

In the UK's water industry the atomic fluorescence measurement coupled to vapour generation techniques has become well established. The use of a hygroscopic membrane drier tube to continuously remove moisture developed by the vapour generator has been particularly useful in laboratory applications [4]. In addition, the range of analytes and concentration levels analysed has been increased using discrete sample injection techniques [5].

Figure 1 shows the layout of the fully automated instrument which can be used to analyse liquid samples. It comprises a random access autosampler, a vapour generator and the Merlin Atomic Fluorescence Detector. These instruments are controlled using an IBM compatible PC.

Figures 2 and 3 show the schematic arrangement of the vapour generator and the transfer of the mercury entrained in an argon carrier gas into the Merlin detector. The switching valve ensures a steady transfer from reagent blank to sample and this minimizes the inherent noise on the signal. A typical signal response for the continuous flow approach is shown in figure 4. The steady state signal is produced by a 10 ppt standard and from this it is easy to show a detection level below 1 ppt without preconcentration. The peak shape is specific to the sample type and the presence of interferents can be recognized should this peak shape deviate from the norm. Table 1 shows a comparison between continuous flow and batch analysis systems. Typically, the measurements can be made over seven orders of magnitude. The discrete sample injection approach allows the system to cope both with high concentrations of mercury; matrix interference effects can also be masked.

0142-0453/96 $12.00 © 1996 Taylor & Francis Ltd.

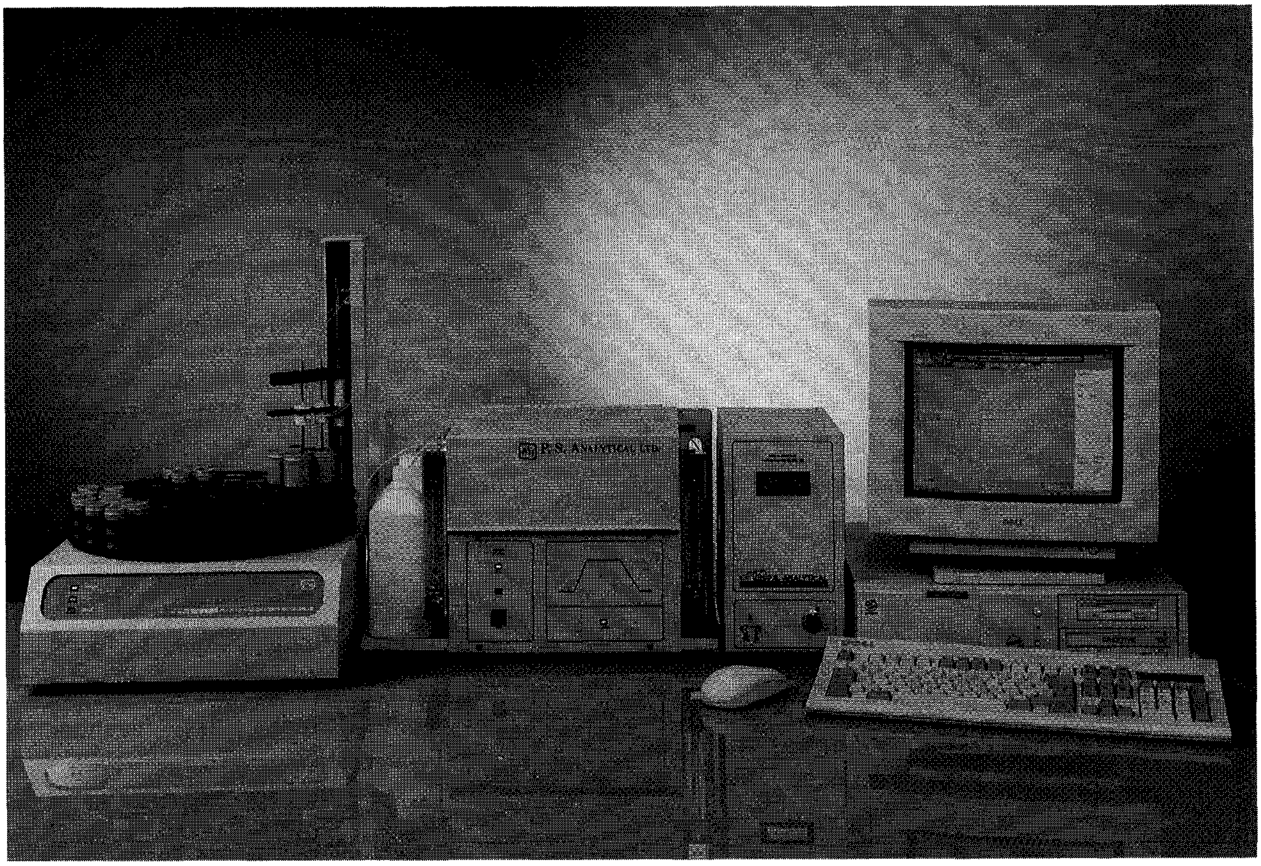

*Figure 1. Merlin Plus system.*

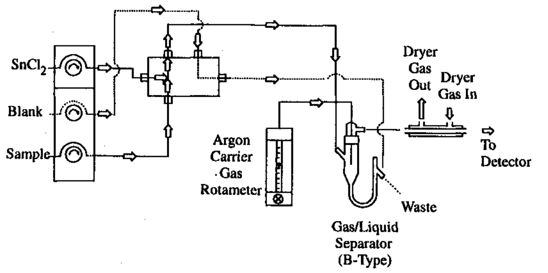

*Figure 2. Hydride valve configuration for sampling.*

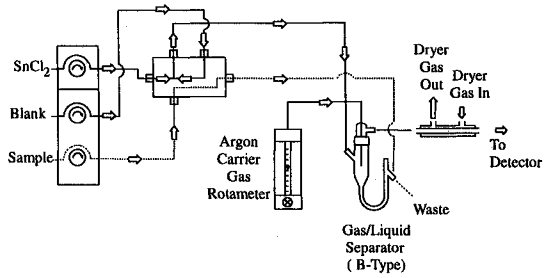

*Figure 3. Hydride valve configuration for blank.*

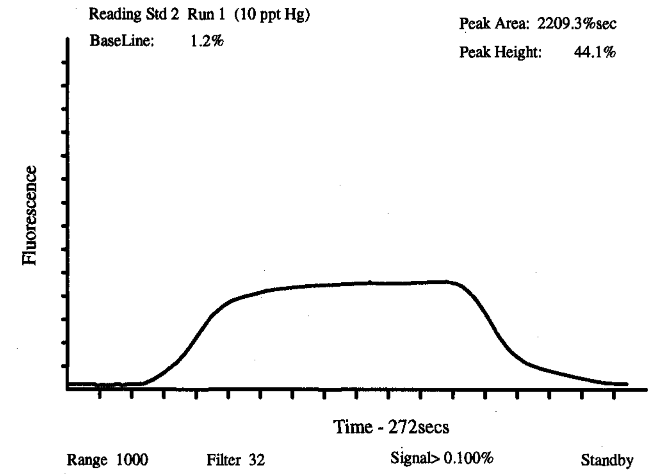

*Figure 4. Typical signal response for the continuous flow approach.*

*Sensitivity*

* Mercury continuous flow. Typical Instrument L.O.D. < 1.00 ppt.

* Further enhancement can be obtained by using the gold trapping technique, with typical improvements of greater than ×10. This is achieved by using the PSA Galahad Pre-concentration Unit.

*Table 1. The advantages and disadvantages of continuous and batch systems.*

| | Continuous flow | Batch analysis |
|---|---|---|
| *Advantages* | Precise control over reaction conditions<br>Constant generation of hydrogen<br>Experienced operators not required<br>Precisions of approx. 1% easily obtainable in linear range | Small sample requirement<br>Economical reagent usage<br>Inexpensive equipment |
| *Disadvantages* | Large sample volume required<br>Long analysis time (60 s) | Operator intensive<br>Precision is function of injection technique<br>Intermittent production of hydrogen<br>Time consuming |

Further reduction of the detect levels has recently been repeated by Cossa *et al.* [6] using an additional concentration step onto a gold/platinum trap. Figure 5 shows the instrumental configuration required for this.

Table 2 sets out the advantages of atomic fluorescence. These basically relate to selectivity and sensitivity, especially the wider linear dynamic range which can extend across many orders of magnitude.

Table 3 sets out the few limitations of the technique. When specifically looking at the situation with mercury these are significantly overcome using the P S Analytical design concepts. The presence of self-absorption at high concentrations and the possibility of matrix effects in, for example, contaminated land samples can be seen as a problem area. This paper shows how the introduction of small discrete samples into the flowing steam can, if properly controlled:

(1) Extend the dynamic range of the analyses.
(2) Effectively eliminate matrix effects by dilution.
(3) Provide the basis of a flexible approach to on-line analyses.

Fluorescence techniques have typical limits of detection below 10 ng $l^{-1}$ with linearity to 100 ng $ml^{-1}$. The linear calibration range stretches over four orders of magnitude which is obviously beneficial in view of the wide range of mercury concentrations found in the environment.

*Table 2. Advantages of atomic fluorescence spectrometry.*

| |
|---|
| Sensitivity attainable is controlled by the intensity of the light source. |
| Equipment can be less complex than that needed for AAS or AES. |
| High sensitivity attainable into the far UV (AAS and AES are insensitive). |
| Good linearity. |
| Low spectral interference. |
| High selectivity. |
| Analytical line summation. |

*Table 3. Disadvantages of atomic fluorescence spectrometry.*

| |
|---|
| Quenching from gaseous species in atom cell. |
| Scattering from light source. |
| Self absorption at high concentrations. |
| Poor sensitivity for elements which absorb and emit in the visible region compared to AES. |

Samples with concentrations exceeding the linearity are susceptible to self-absorption. This process is best explained using a standardized fluorescence cell like that shown in figure 6.

This theoretical model assumes that the light beams are parallel and that there is uniform atomic concentration and temperature. At high concentrations, incident radiation passing through Δl may be lost by absorption before excitation can occur. Useful fluorescence may also be lost by reabsorption in the region ΔL. In an ideal situation these regions would be infinitely small, thereby minimizing self-absorption. Figure 7 shows a typical profile obtained using the continuous flow approach for a 2000 μg $l^{-1}$ mercury solution and the self-absorption process is clearly evident. As the concentration increases, there is a rapid rise in signal until the concentration has reached a level where self-absorption occurs. At this point the signal begins to fall, in severe cases to zero. When the sample is removed the concentration begins to decline and the signal begins to rise once more. Carry-over times between samples can be up to 5 minutes depending on the concentration of mercury present.

The atomic fluorescence signal magnitude can be reduced with the use of alternative carrier gases, such as nitrogen or air. These gases have been found to reduce the fluorescence signal by eight and 30 times, respectively, due to quenching. This is basically radiation-less deactivation of excited atoms due to collisions with foreign species present in the cell. The effectiveness of this process is dependent on the rate at which collisions occur, the type of non-radiative process involved and the effective cross-section of the quenching species. The fraction of absorbed photons actually re-emitted as fluorescence radiation is known as the fluorescence yield, $\phi$.

This is defined as:

$$\phi = \frac{B_{ij}}{\text{Total probability per second of de-excitation}}$$

where $B_{ij}$ is the Einstein coefficient for fluorescence emission. The total probability of de-excitation is the summation of $B_{ij}$ with the rate of all non-radiative processes contributing to quenching. The quenching process occurring with mercury in the presence of nitrogen or air is due to inelastic collisions involving transfer of energy. The process for nitrogen is thus:

$$Hg^* + N_2 \rightleftharpoons Hg + N_2$$

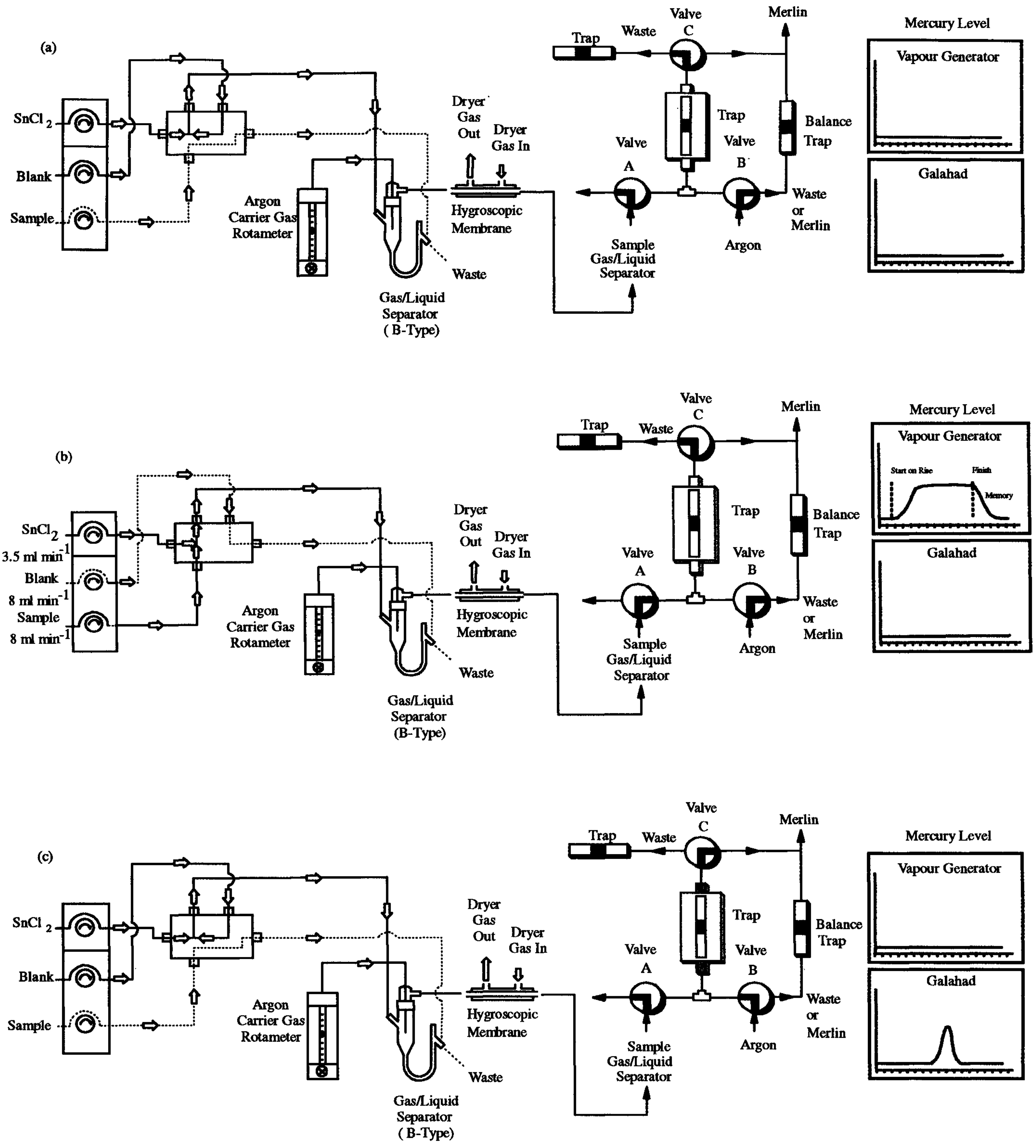

*Figure 5. Instrumental configuration.*

where the superscript * is used to indicate the excited state. The rate, $r$, of each collision is defined as the number of excited mercury atoms quenched per second per unit volume and can be expressed in the form:

$$r = k[Hg^*][N_2]$$

where $k$ is the rate constant for the process. The probability of an excited mercury atom being quenched is therefore $r/[Hg^*]$. Hence the fluorescence yield factor for mercury with quenching caused by nitrogen will be:

$$\phi_{253.7} = \frac{B_{10}}{k[N_2] + B_{10}}$$

where $B_{10}$ is the Einstein coefficient for the excited state to the ground state transition. It therefore follows that the maximum value of $\phi$ is unity where no quenching occurs. This, however, is unlikely to occur.

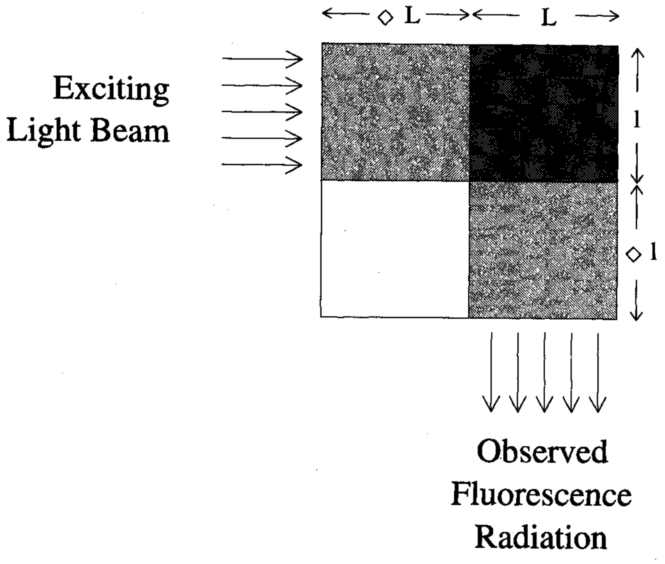

*Figure 6. Standardized fluorescence cell.*

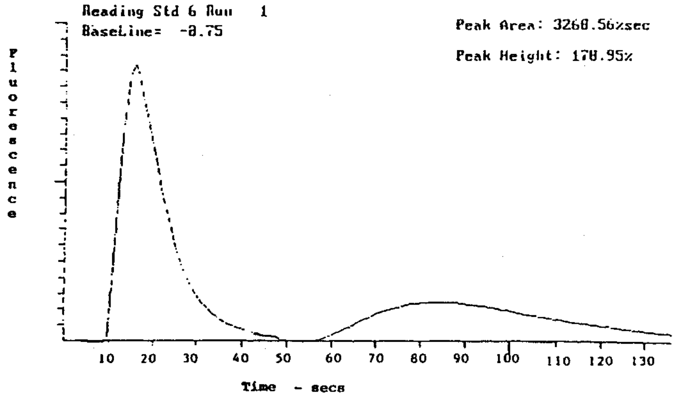

*Figure 7. Typical peak shape, illustrating the process of self-absorption, using the continuous-flow approach for a 2000 μg $l^{-1}$ solution of mercury.*

Although a reduction in signal is clearly observed, the quenching process has no relation to linearity because the self-absorption process is dependent on the atomic concentration and the atom cell dimensions. The reduction in signal from quenching therefore has no practical use in this application. The analytical response curve for argon and nitrogen is shown in figure 8 for continuous flow vapour generation.

Discrete sample analysis typically uses volumes between 50 and 200 μl. Although not as sensitive as the continuous flow approach, it is less suceptible to self-absorption and matrix interference. A schematic arrangement for a discrete sample analyses is shown in figure 9. This approach has been subsequently superseded by using the standard P S Analytical vapour system configuration for the 10.004 model. With this instrument all the time cycles of the vapour generator are programmed by the computer software. The discrete volume is therefore determined as a fraction of flow rate and the time of valve opening. The limitation on this effect is the dead volume within the switching value itself. This allows the upper limit of the calibration range to be increased. Figure 10 shows three analytical response curves corresponding to 75, 100 and 200 μl loop sizes. The smaller volumes gave higher upper limit calibration ranges, with slightly less sensitivity. An estimation of the sensitivity is again obtained from the slope of the curve at the point where deviation from linearity occurs. Table 4 summarizes the effect of sample volume on linearity.

Samples containing levels of mercury exceeding the linear range are still susceptible to self-absorption; a typical profile is shown in figure 11. The profile corresponds to a 100 000 μg $l^{-1}$ solution of mercury, and the self-absorption is clearly observed. However, this is not as severe as that for continuous flow and the carry-over times between samples with high levels is negligible. This allows the analysis of total samples to proceed with minimal delay.

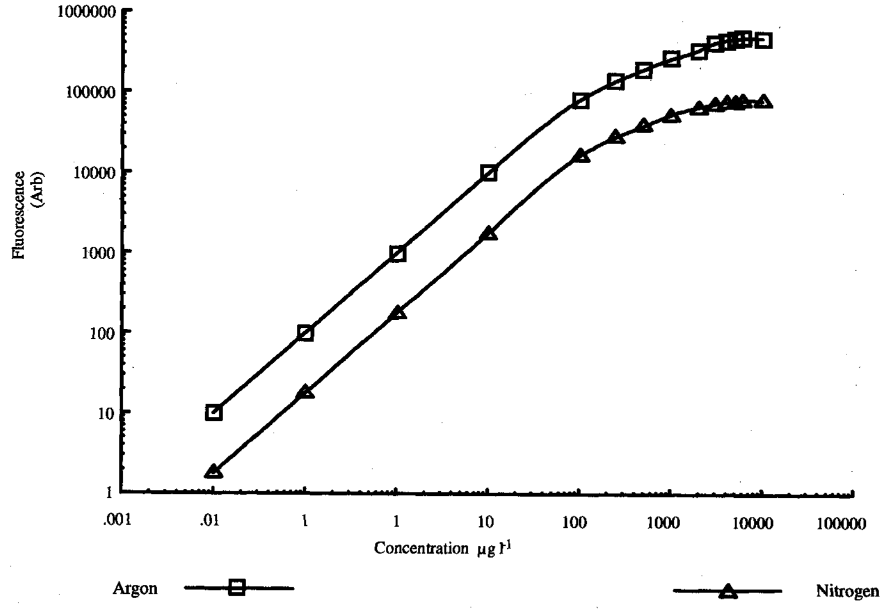

*Figure 8. Analytical response curves for continuous-flow atomic fluorescence spectrometry using both argon and nitrogen carrier gases.*

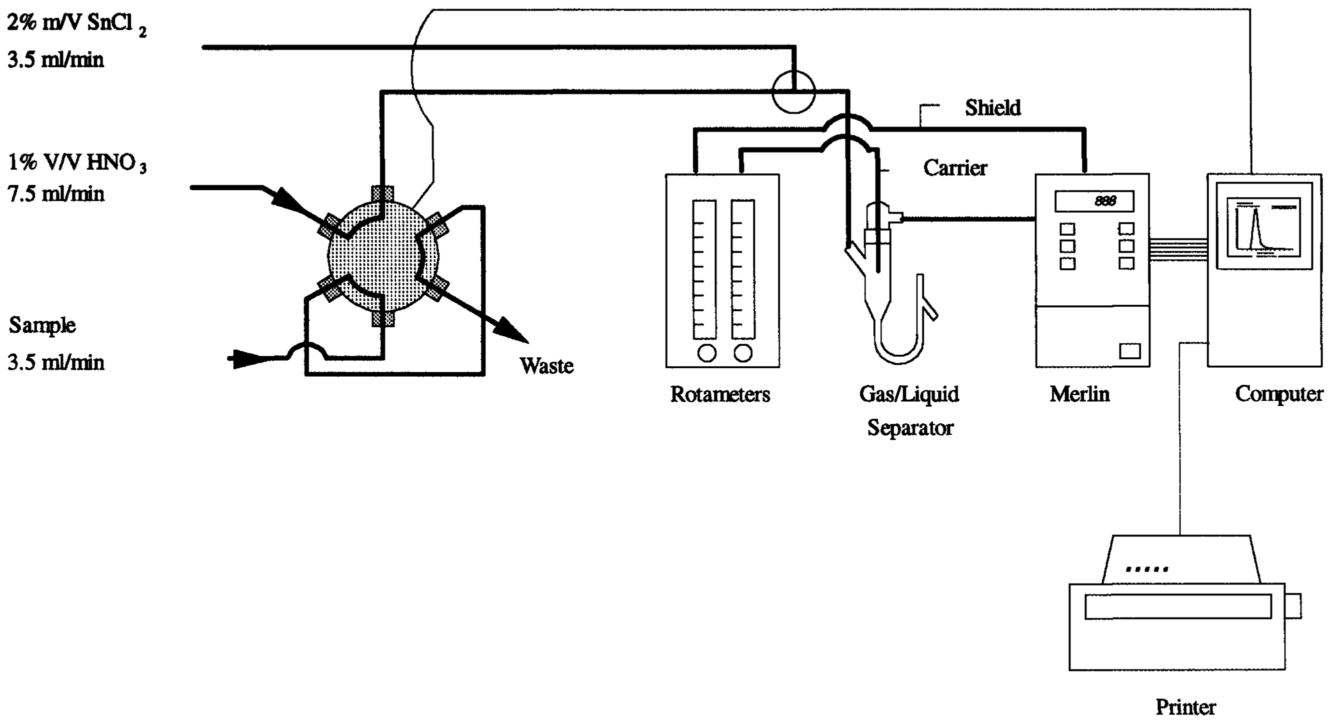

*Figure 9. Schematic arrangement for a discrete sample analysis.*

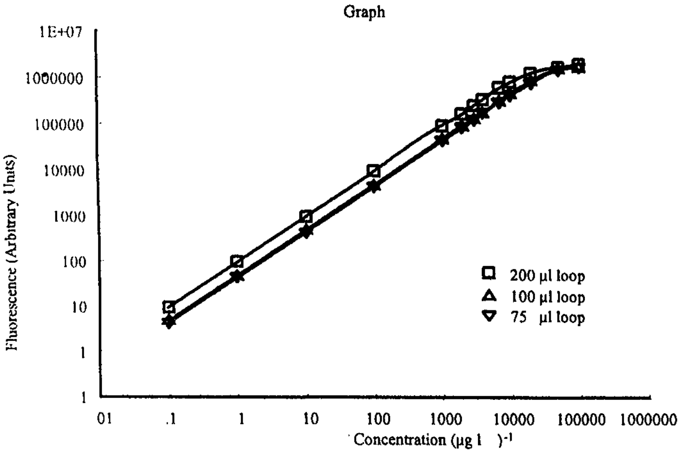

*Figure 10. Three analytical response curves corresponding to 75, 100 and 200 μl loop sizes.*

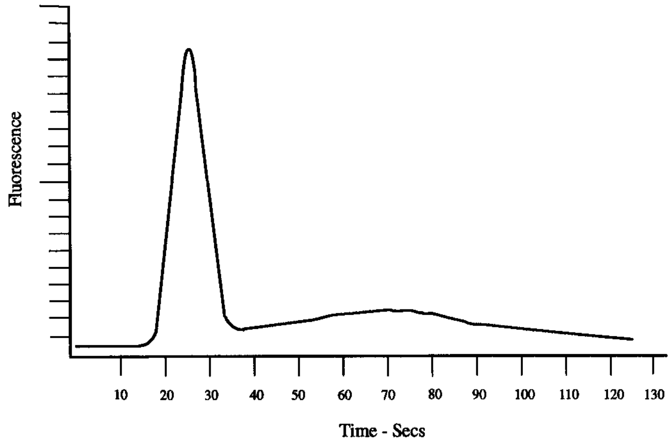

*Figure 11. Typical peak shape, illustrating the process of self-absorption, for the flow-injection approach for a 100 000 μg $l^{-1}$ solution of mercury.*

*Table 4. The effect of sample volume on linearity.*

| Sample volume (μl) | Upper limit calibration range (mg $l^{-1}$) | Slope |
|---|---|---|
| 75 | 10·5 | 3·7 |
| 100 | 10 | 4·4 |
| 200 | 7 | 8·4 |

To assess the validity of the flow-injection cold vapour-atomic fluorescence spectrometry (CV-AFS) technique, a range of certified reference materials and zinc battery anodes has been analysed for mercury. These results are shown in table 5. Table 5 shows that accurate, precise quantitative measurements can be made using the

*Table 5. Determination of mercury in certified reference materials and battery anodes.*

| Certified reference material | Expected/certified concentration | Concentra found | Weight dilution |
|---|---|---|---|
| NIST SRM 1641b (mercury in water) | 1·52 ± 0·04 (μg $ml^{-1}$) | 1·41 ± 0·04 (μg $ml^{-1}$) | 0 |
| NBS SRM 3133 (spectrometric solution) | 10·00 ± 0·01 (μg $ml^{-1}$) | 9·89 ± 0·20 (μg $ml^{-1}$) | 2500 |
| Zinc Anode A | 1000 (μg $ml^{-1}$) | 1060 ± 30 (μg $g^{-1}$) | 200 |
| Zinc Anode B | 0 | 4·11 ± 0·29 (μg $g^{-1}$) | 200 |
| Zinc Anode C | 1200 (μg $ml^{-1}$) | 1150 ± 43 (μg $g^{-1}$) | 200 |

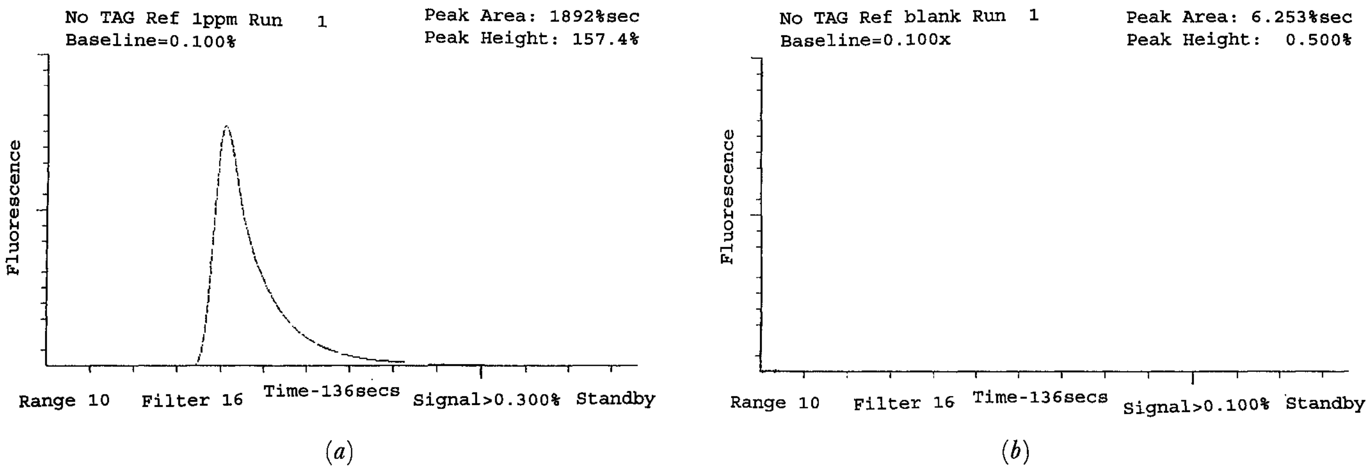

*Figure 12. Analysis of 1 ppm followed immediately by blank—the signal trace for this is running along the baseline. Levels higher than 1 ppm will automatically be reset to blank when the software senses that the signal level will go off-scale.*

flow-injection CV-AFS approach. The advantage of this system is that minimal sample dilution is required, which considerably reduces the sample preparation time and errors involved in large serial dilutions. One further advantage is that matrix interference is reduced because the analyte is separated from the matrix by generation of the gas and because small volumes are utilized.

Another major advantage of the discrete approach is that there is little interference or carry-over from one sample to another. This allows linear calibrations up to 10 ppm (as shown in table 4). However, it is possible to analyse samples up to 100 ppm with little or no carry-over between the high and low sample. The signal from 100 ppm will provide a detector overload, but the selection valve in the vapour generator will quickly switch to the blank/standby situation thereby returning the signal to the baseline. The next sample to be analysed can be accurately determined. Figure 12 illustrates this point with results for a sample greater than 100 ppm followed by a blank.

The application of the discrete sample injection and the capabilities of the continuous flow approach can be effectively married together using the method chaining approach developed by P S Analytical. The detector has a pre-amplifier allowing selection of gain ranges between 1 and 1000. In standard operation mode, the gain range is pre-selected for different concentration ranges. One gain range will allow a calibration span of two orders of magnitude. This provides the most accurate and precise methods of analysis. Samples which contain concentrations above the calibration range are normally diluted manually after the analytical run. The method chaining facility allows different methods with different gain ranges to be changed together, so that no manual dilutions for samples that are above the calibration range are required.

The discrete sample mode can be used to assign samples to the appropriate calibration ranges. With a sampling rate of 80 samples per hour, the samples can be quickly screened to estimate levels. The reproducibility of the discrete injection mode is illustrated in figure 13, which shows replicate analyses in the ppb region for a mercury standard solution containing 1 $\mu g\, l^{-1}$.

In method chaining, up to five different methods, each with a unique calibration, can be coupled together. This can be illustrated by reference to two calibration methods. In the first, the calibration range is set between 0–1 $\mu g\, l^{-1}$; if samples above 1 $\mu g\, l^{-1}$ are analysed offscale, recognition will switch the solution to the reagent blank, thus conserving the sample and minimizing carry-over. After the autosampler programme is complete the system is recalibrated at a higher range, such as 0–100 $\mu g\, l^{-1}$ and these samples, which previously went offscale, are re-analysed. All conditions on the vapour generator are set automatically by the software programme. The operational sequence of the method chaining is activated from the autosampler programming mode.

### On-line applications

Discrete sampling has a major advantage when dealing with complex matrices, especially with concentrated acids or alkalis. The effective dilution step has been extremely beneficial when combined with the extremely low detection capabilities of the detector to analyse such samples. For on-line process analyses this benefit has further advantages: sample volume is reduced; the risk of contamination between corrosive materials and the instrumentation is minimized; and the response time to changes in sample concentration is also reduced.

In comparison to laboratory analyses, the chemistry involved must be more complex in order to cope with the digestion of all forms of mercury to mercury(II), prior to the tin(II) chloride reaction. The authors have directed their research to a number of chemical regimes, but in this paper reference is only made to the application to concentrated sulphuric acid.

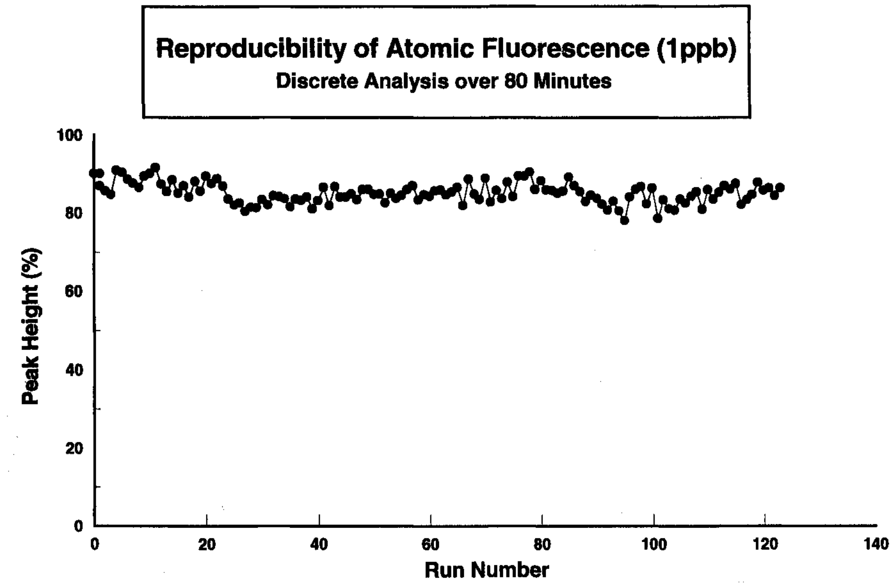

*Figure 13. Response curve—the method chaining facility allows the user to run up to five different analytical methods in sequence.*

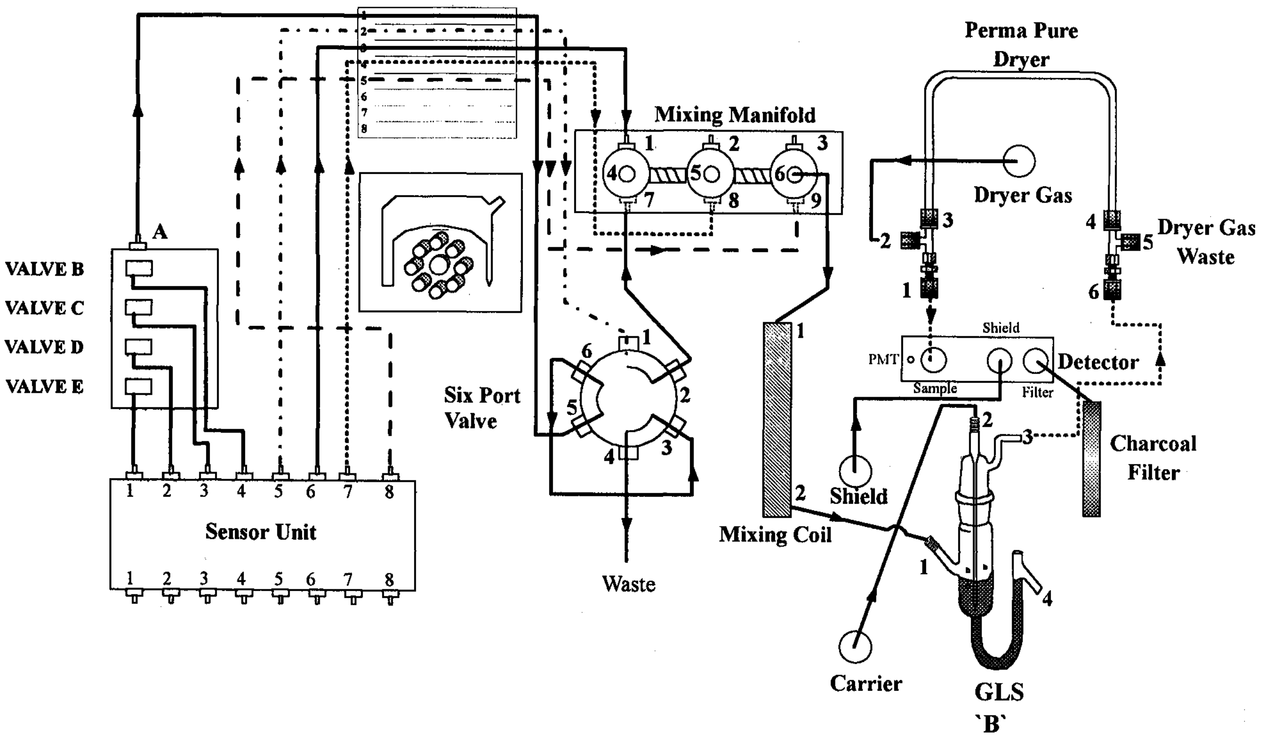

*Figure 14. Schematic arrangement of the chemical manifold required for the determination of mercury at low levels in concentrated sulphuric acid.*

Table 6 shows the specific considerations that require attention prior to translating a laboratory instrument to on-line applications. Reagent consumption is a prime consideration since it is desirable that little, if any, maintenance is required at rates greater than one week.

Figure 14 shows the schematic arrangement of the chemical manifold required for the determination of mercury at low levels in concentrated sulphur acid developed by Brahma *et al.* [7]. The oxidation or digestion

*Table 6. Specific considerations required to translate laboratory instrumentation to process applications.*

| |
|---|
| Conversion of all mercury species to divalent mercury. |
| Low reagents consumption and reagent stability. |
| Stable and rugged detection system. |
| Reliable interface between sample stream and on-line system. |
| Fault diagnostics with feedback system. |
| Data processing via CPUs. |

```
Changed Hg by Online A.F.S. Hg 3 Stds to 1.086ppm
 Method   | Signal | Sampler | Tag    | Reference  | Done | Runs | 7 Jun 96
 HGONLIN1 | -0.50% | None    | SAMPLE | nointerfer | 244  | 1    | 14:35:22
 Library  Method  Calibrate  Options  Analyse  Results  Setup  TouchStone

Method: Hg by Online A.F.S.
Curve Fit : Least Squares Straight Line
Measured by : Peak Ht

     Flow Injection Valve Setup

 DELAY          MEASURE          RESET

 130sec         200sec           100sec

 Loop: 100ul         Fill Rate: 1 ml/min

PSA Merlin Calibration Setup:

RANGE 10        FINE 10.0     ZERO Auto

Range & Running 1/4s set automatically

  SENSOR

1: Blank                 2: Low Standard

3: High Std              4: Sample

5: 20% HCL               6: 0.1 Br/BrO3

7: 1%OHNH3Cl             8: 2% SnCl2

N.B.All Pump Lines RED-RED

        F1-Help  F2-Keep Displayed  F3-Print  F4-Edit  Esc-Menu

```

*Figure 15. Methods page from the computer software.*

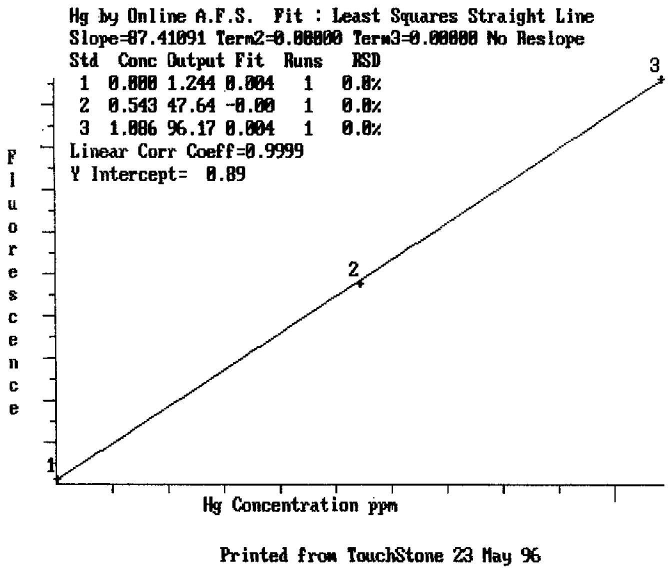

*Figure 16(a). Simple response for a repeat sampling sequence.*

step is provided by the reaction of potassium permanganate. This is then followed by a conventional tin II chloride reduction to form mercury vapour which is introduced into the detector. Figure 15 shows the methods page from the computer software. To minimize matrix inteference effects and excessive heat generation, the system is used in a discrete sampling mode. Reagent flow rates are kept at a level of 0·5 ml min to conserve reagents and maximize the time between reagent changes in the instrument. The analytical cycle performed is to analyse a blank, a 100 ppb standard, a 200 ppb standard and then the sample stream four times prior to repeating the analytical sequence to fit the analytical needs at the time. The inherent sensitivity of the atomic fluorescence detector allows air (which provides a 30-fold quenching of the signal) to be used as a transfer gas. For process applications, this provides the site managers with a considerable comfort factor because the dangers of asphyxiation due to other carrier gases are overcome.

The analysis is operated in a similar manner to the laboratory instrument, which is somewhat different to conventional laboratory analyses. Repeat cycle discrete analyses are performed using the software facilities to continuously update the calibration and blank values; this means that the results are continuously updated. Figure 16 shows a simple response for a repeat sampling sequence and a calibration graph over a 48-hour period.

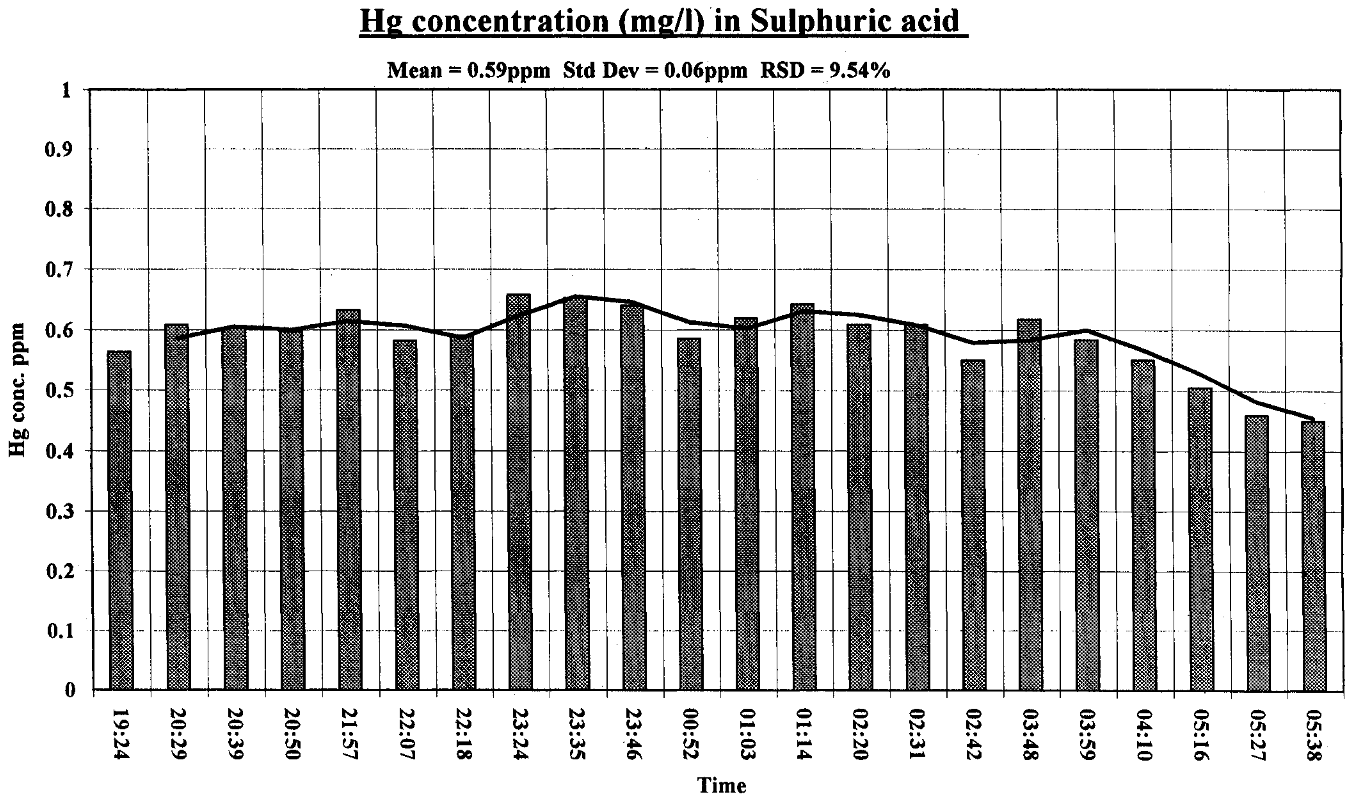

*Figure 16(b). Calibration graph over a 48-hour period.*

### Conclusions

The sensitivity of the continuous flow vapour generation system coupled to atomic fluorescence provides extremely low detection levels. The combination with discrete sample introduction extends the linear dynamic range of the instrumentation to encompass seven orders of magnitude. In addition, this mode of operation extends the capabilities of the system to handle complex matrices and also to provide extremely versatile on-line process instrumentation.

### References

1. Jones, R., Jaffe, R. and Azaam, A., *Journal of High Resolution Chromatography*, **17** (1994), 745.

2. Thompson, K. C. and Godden, R. G., *Analyst*, **100** (1975), 544.

3. Godden, R. G. and Stockwell, P. B., *Journal of Analytical Atomic Spectrometry*, **4** (1989), 301.

4. Corns, W. T., Ebdon, L. C., Hill, S. J. and Stockwell, P. B., *Analyst*, **117** (1992), 717.

5. Corns, W. T., Ebdon, L. C., Hill, S. J. and Stockwell, P. B., *Journal of Automatic Chemistry*, **13** (1991), 267.

6. Cossa, D., Sanjuan, J., Cloud, J., Stockwell, P. B., and Corns, W. T., *Journal of Analytical Atomic Spectrometry*, **10** (1995), 287.

7. Brahma, N., Corns, W. T., Evans, E. H. and Stockwell, P. B., Private communication.

